# Decoding the NRF2–NOTCH Crosstalk in Lung Cancer—An Update

**DOI:** 10.3390/antiox14060657

**Published:** 2025-05-29

**Authors:** Angelo Sparaneo, Filippo Torrisi, Floriana D’Angeli, Giovanni Giurdanella, Sara Bravaccini, Lucia Anna Muscarella, Federico Pio Fabrizio

**Affiliations:** 1 Laboratory of Oncology, Fondazione IRCCS Casa Sollievo della Sofferenza, 71013 San Giovanni Rotondo, Italy; a.sparaneo@operapadrepio.it; 2 Department of Drug and Health Sciences, University of Catania, 95123 Catania, Italy; filippo.torrisi@unict.it; 3 Department of Medicine and Surgery, University of Enna “Kore”, 94100 Enna, Italy; floriana.dangeli@unikore.it (F.D.); giovanni.giurdanella@unikore.it (G.G.); sara.bravaccini@unikore.it (S.B.)

**Keywords:** oxidative stress, NRF2, NOTCH, lung cancer, resistance

## Abstract

The Nuclear factor erythroid 2-related factor 2 (NRF2) Neurogenic locus NOTCH homolog protein (NOTCH) crosstalk has emerged as a critical regulatory axis in the progression of solid cancers, especially lung, affecting tumor growth and resistance to therapy. NRF2 is a master transcription factor that orchestrates the cellular antioxidant response, while NOTCH signaling is involved in the cell–cell communication processes by influencing the patterns of gene expression and differentiation. Although frequently altered independently, genetic and epigenetic dysregulation of both NRF2 and NOTCH pathways often converge to deregulate oxidative stress responses and promote tumor cell survival. Recent findings reveal that the NRF2/NOTCH interplay extends beyond canonical signaling, contributing to metabolic reprogramming and reshaping the tumor microenvironment (TME) to promote cancer malignancy. Emerging scientific evidences highlight the key role of biochemical and metabolomic changes within NRF2–NOTCH crosstalk, in contributing to cancer progression and metabolic reprogramming, beyond facilitating the adaptation of cancer cells to the TME. Actually, the effects of the NRF2–NOTCH bidirectional interaction in either supporting or suppressing lung tumor phenotypes are still unclear. This review explores the molecular mechanisms underlying NRF2–NOTCH crosstalk in lung cancer, highlighting the impact of genetic and epigenetic deregulation mechanisms on neoplastic processes, modulating the TME and driving the metabolic reprogramming. Furthermore, we discuss therapeutic opportunities for targeting this regulatory network, which may open new avenues for overcoming drug resistance and improving clinical outcomes in lung cancer.

## 1. Introduction

Nuclear factor erythroid 2-related factor 2 (NRF2) and Neurogenic locus NOTCH homolog protein (NOTCH) are master regulators of signaling pathways that orchestrate several biological and cellular behaviors as well as cell proliferation, self-renewal, differentiation, and response to physical and chemical insults [1]. NOTCH1–NOTCH4 receptors belong to single-pass trans-membrane family proteins that are activated by canonical ligands belonging to the Delta-like and Serrate/Jagged families. They are essential for the determination of cell fate and the maintenance of homeostasis in multiple tissues and organs [2]. However, in solid cancers, NOTCH signaling can be considered as a two-sided coin, since, on the one hand, it contributes to guide tumor growth but, on the other hand, it has been reported that its oncogenic properties could switch towards a suppressor role where cell proliferation and survival are strongly limited by cellular context [3].

The transcription factor NRF2, encoded by *NFE2L2* gene, exerts a protective activity in counteracting the accumulation of reactive oxygen species (ROS). These ROS are generated during mitochondrial oxidative phosphorylation and in response to xenobiotics [4,5]. Under cellular stress, NRF2 dissociates from the binding with its negative regulator Kelch-like ECH-associated protein 1 (KEAP1), moves into the nucleus and activates the transcription of several cytoprotective genes through the interaction with their antioxidant response elements (AREs) [6]. In an oncogenic context, the aberrant activation of NRF2 leads to elevated oxidative stress tolerance, enhanced proliferation, tumor progression, and altered metabolic reprogramming, that collectively contribute to resistance against chemotherapy and radiotherapy [7]. It would be interesting to investigate whether an increased efficacy of chemo- and radiotherapy and a reduced lung cancer cell resistance may arise from dual pharmacological targeting of NRF2 and NOTCH pathways, so as to represent a promising therapeutic strategy in preclinical and clinical studies [8,9].

Over the past nine years, critical progress has significantly reshaped the understanding of NRF2–NOTCH crosstalk in lung cancer. First, new mechanistic insights revealed that NRF2 directly activates NOTCH1 transcription by binding antioxidant response elements (AREs) within its promoter, establishing a stress-responsive transcriptional axis [10]. Conversely, canonical NOTCH targets such as Hairy and Enhancer of Split 1 (HES1) and Hairy/Enhancer-of-split related with YRPW motif protein 1 (HEY1) have been shown to modulate NRF2 target gene expression, forming a functional feedback loop that coordinates redox homeostasis with proliferation and cell differentiation [11]. This bidirectional regulatory architecture was not discussed in our prior review [12]. Second, recent work has uncovered a major role of epigenetic modulation—such as aberrant promoter methylation of *NFE2L2* and *NOTCH* genes or histone remodeling via Enhancer of Zeste Homolog 2 (EZH2) and Histone Deacetylases (HDACs)—in fine-tuning NRF2–NOTCH signaling output, thus linking chromatin state to redox and developmental signaling plasticity [13]. Third, the metabolic dimension of the NRF2–NOTCH interplay has emerged as a key node of convergence, with coordinated regulation of glycolysis, the pentose phosphate pathway, and glutaminolysis contributing to metabolic reprogramming, NADPH production, and antioxidant defense [14]. The increasing evidence in this field significantly expands the know-how on functional NRF2–NOTCH crosstalk and connects to hallmark features of tumor progression and treatment resistance.

Finally, in terms of scope, the present manuscript offers a substantial and systematic expansion beyond our previous review. The reported and updated literature includes critical findings on emerging regulatory layers and crosstalk mechanisms, not previously addressed [12]. While our prior review focused predominantly on genetic and transcriptional aspects of NRF2 and NOTCH signaling, the present work increases the coverage to include: (1) epigenetic deregulation, not only related to DNA methylation but also included histone acetylation/methylation patterns, non-coding RNAs (ncRNAs) and long non-coding RNAs (lncRNAs) that modulate NRF2–NOTCH signaling [15]; (2) tumor metabolism, describing how NRF2-driven glycolytic flux and glutaminolysis interact with NOTCH-mediated metabolic shifts under hypoxia [16]; (3) post-translational modifications including phosphorylation, ubiquitination events that fine-tune the stability, localization, and activity of NRF2 and NOTCH proteins (e.g., KEAP1-Cullin 3 (CUL3)-NRF2 axis) [17].

By integrating these multi-layered regulatory mechanisms [18], here we present a more comprehensive genetic, epigenetic, and metabolic evidence underlying NRF2 and NOTCH signaling, underlining the importance of this crosstalk as a potential molecular target to provide new insights into therapeutic strategies for patients with lung tumors.

## 2. NRF2 Signaling: From Physiological Guardian to Oncological Role

Physiologically, cells respond to stress by activating molecular and energy-related processes to restore homeostasis, increasing catabolic activity and oxidative phosphorylation, which leads to ROS accumulation and oxidative stress [5,19]. To survive, tumor cells exploit antioxidant systems linked to enzymes like superoxide dismutase (SOD), glutathione peroxidase (GPx), and catalase (CAT), while hijacking transcription factors, including Basic Leucine Zipper (bZIP) domain proteins, named for the basic amino acid residues in the alpha-helical regions, to regulate gene expression and sustain malignant growth [6,20].

**Figure 1:**
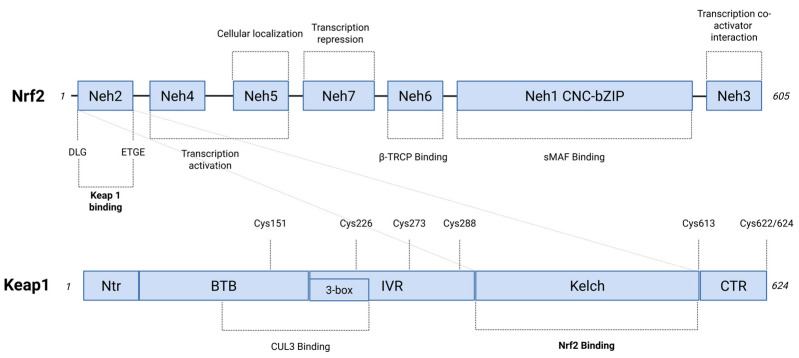
Functional NRF2 and KEAP1 proteins domains. On the top, NRF2 is a 605 amino acid protein that consists of seven functional domains (Neh1–Neh7), each of which leads to the modulation of both NRF2 transcriptional activity and protein stability. KEAP1-binding region is located within the amino-terminal Neh2 domain that contains Asp-Leu-Gly (DLG) and Glu-Thr-Gly-Glu (ETGE) motifs. The β-transducin repeat-containing protein (β-TRCP) interaction occurs in the Neh6 domain. The binding to sMAF (small musculoaponeurotic fibrosarcoma) transcription factors takes place through the Neh1 domain. This interaction affects Cap ‘n’ Collar basic leucine zipper (CNC-bZIP) motif, which is responsible for mediating the binding of AREs (antioxidant response elements). The figure includes the functional roles from Neh1 to Neh7 domains. On the bottom, five domains represent the structure of the KEAP1 protein (624 amino acids residues): Ntr (the N-terminal region), Broad complex, Tramtrack, and Bric-a-brac (BTB), intervening region (IVR), double glycine repeat (DGR) that consists of six kelch repeats, which is involved in the binding sites that interact with NRF2 (Kelch), CTR (C-terminal region). The BTB and IVR domains are involved in Cullin 3 (CUL3) binding, leading to the formation of E3 ubiquitin ligase complex. A dashed line indicates the KEAP1 stress-sensing function by linking the three regions that define the H_2_O_2_ stress sensor center (Created with BioRender.com, Licensing and Agreement number EZ285EVYX5, accessed on 15 April 2025).

A subfamily of these transcription factors, the Cap ‘n’ Collar (CNC) proteins, possesses a 43-amino acid conserved region (CNC domain) at the N-terminal portion [21]. One of the main transcription factors within the CNC-Basic Leucine Zipper (bZIP) protein family is NRF2, which serves as a master regulator of stress responses under physiological condition. Cancer cells can manipulate the NRF2-KEAP1 cellular pathway to survival and progress [22]. In response to endogenous or exogenous stressors, NRF2 forms a transcriptional complex with its direct heterodimeric partner, small Maf (sMaf), and co-factors such as CREB-binding protein (CBP)/p300. This complex binds to numerous ARE of genes, implicated in cellular adaptation and homeostasis maintenance [23]. The NRF2 pathway is finely regulated by a ubiquitination system that operates under normal cellular homeostasis when an active stress response is unnecessary [24]. The proteasomal ubiquitination system continuously degrades NRF2 by polyubiquitination of NRF2 at seven key lysine residues via KEAP1, an adaptor protein of CUL3–ring-box 1 (RBX1)-containing E3 ubiquitin ligase complex [25] (Figure 1).

The activity of NRF2 is regulated by two primaries KEAP1-dependent and KEAP1-independent pathways [6,26]. In the KEAP1-dependent pathway, thiol-reactive compounds, such as ROS, induce conformational changes in KEAP1, preventing NRF2 ubiquitination. These conformational changes are facilitated by sensor amino acids such as cysteine residues of KEAP1, which undergo modifications upon reacting with ROS [24]. The accumulation of NRF2 in the cytoplasm promotes its translocation into the nucleus, where it functions as a transcription factor by binding to AREs of several genes [27]. Once homeostasis is restored, NRF2 is exported back to the cytosol via KEAP1, which enters the nucleus through karyopherin alpha 6 (KPNa6), [28]. Additionally, KEAP1 itself can be subjected to ubiquitination. Under stress conditions, a key regulator of this process is Sequestosome 1 (p62), which binds to KEAP1, thereby disrupting its interaction with NRF2. This interaction facilitates KEAP1 degradation via the autophagy–lysosomal pathway, mediated by the vesicular protein complex LC3 [6]. KEAP1-independent NRF2 regulation involves proteins that compete with KEAP1 for NRF2 binding, as protein kinases that modulate NRF2 interactions with the ubiquitination complex [6]. Other transcription factors, such as nuclear factor κ-light-chain-enhancer of activated B cells (NF-κB), may also play a role in NRF2 regulation, although the precise interconnections between these pathways remain unclear [29].

Notably, KEAP1 has been shown to bind to Inhibitory kappa B kinase beta (IKKβ), promoting its ubiquitination and degradation [30]. Additionally, the aryl hydrocarbon receptor (AHR) transcription factors contribute to KEAP1-independent NRF2 regulation by activating both ARE and xenobiotic-responsive elements (XREs) found in proximity within the promoters of several NRF2-regulated genes, including NRF2 itself [31]. Beyond these mechanisms, many post-transcriptional regulatory processes, such as microRNA (miRNA)-mediated regulation, phosphorylation, and acetylation, can also influence NRF2 activity, contributing to its nuclear exclusion and degradation [32].

Given its ability to protect cells, NRF2 plays a dual role in cellular homeostasis [33]. While it serves as a guardian of cellular defense under normal conditions, it can also transition into a proto-oncogene when hijacked by tumor cells, contributing to treatment resistance [34]. NRF2 is a key regulator of antioxidant response and cellular detoxification, mediating the transcription of genes such as glutamate–cysteine ligase catalytic/modifier subunit (GCLC/M) and glutathione S-reductase (GSR), which are essential for glutathione synthesis and recycling [35]. It also regulates heme oxygenase-1 (HO-1), peroxiredoxin-1/4, superoxide dismutases (SODs), thioredoxin (TRX), thioredoxin reductase 1 (TRXR1), and aldo-keto reductases (AKRs), all of which play crucial roles in detoxifying xenobiotic compounds [36]. Studies on *NRF2-knockout* mice have demonstrated the importance of this pathway, revealing an absence of cytoprotective effects, increased tissue damage, and heightened carcinogenesis [22].

However, the loss of NRF2’s protective function is not solely due to its exploitation by tumor cells [37]. Dysregulation of the pathway at the physiological level leads to alterations in the cellular microenvironment, which, when chronically exposed to stress factors, undergoes aging processes that may culminate in tumorigenesis [38]. Persistent inflammation drives oxidative stress, which in turn disrupts NRF2 homeostasis [24,39]. This persistent activation can result in sustained pathway upregulation, promoting the survival of precancerous lesions that would otherwise undergo elimination, and thereby contributing to tumor initiation and progression [40].

In addition to external factors, genetic and epigenetic alterations also play a role in converting *NFE2L2* into a proto-oncogene. In this context, NRF2 intersects key oncogenic pathways, including Kirsten rat sarcoma viral oncogene homolog (KRAS), v-raf murine sarcoma viral oncogene homolog B1 (BRAF), and MYC (c-myc), and provides protection from ROS and sustains the reducing environment necessary for anabolic metabolism in proliferating cells [41]. The phosphoinositide 3-kinase (PI3K)/protein kinase B (AKT) pathway, loss of Phosphatase and tensin homolog (PTEN), and Mammalian Target of Rapamycin (mTOR) signaling also work in concert with NRF2 to promote tumor growth [42]. The interaction of NRF2 with estrogen receptors also contributes to the dual role of NRF2 in cellular processes. The interplay between estrogens and NRF2 suggests a cytoprotective effect mediated by female hormones [43]. Studies have shown that 17β-estradiol (E2) enhances cell viability, reduces ROS production, activates Akt signaling, and inhibits glycogen synthase kinase-3β (GSK-3β), leading to increased NRF2 activity [44]. This results in upregulated HO-1 expression and SOD activity, which mitigate the neurotoxic effects of homocysteine [45]. In mouse embryonic fibroblasts (MEFs), E2 treatment induces nuclear translocation of NRF2 enhancing tumor necrosis factor (TNF)-α-induced NF-κB activation and upregulates its downstream target, inducible nitric oxide synthase (iNOS) [46]. Alternatively, tumor cells can exploit this mechanism to enhance their defenses against oxidative stress, thereby improving survival and proliferation [47]. Moreover, 17β-estradiol cooperates with ER-α36- Epidermal Growth Factor Receptor (EGFR) signaling, further amplifying NRF2 activation and promoting cell proliferation [44]. This interaction can activate the Ras/PI3K/PTEN/Akt pathway, which enhances NRF2 nuclear translocation and stability, ultimately contributing to tumor progression [42].

Finally, NRF2 dysregulated may contribute to tumorigenesis by suppressing apoptosis and promoting cancer cell survival via the tumor protein 53 (p53), often referred to as the “guardian of the genome” due its crucial role in maintaining genomic integrity [48,49]. NRF2 can influence p53 regulation through the induction of Mouse double minute 2 homolog (MDM2), which in turn inhibits p53 activity [37].

## 3. Genetic and Epigenetic Mechanisms of KEAP1 and NFE2L2 and Their Role in Lung Cancer Development and Progression

Somatic mutations of *KEAP1* and *NFE2L2* genes are frequent in Non-Small-Cell Lung Cancer (NSCLCs), and show specific molecular pattern linked to different histological subtypes of lung cancer [50]. More specifically, and as reported in many other scientific contexts, The Cancer Genome Atlas Network (TCGA) reported the recurrence of these mutations in lung adenocarcinomas (LUADs, 23% of cases) and in lung squamous cell carcinomas (LUSC, 34% of cases), respectively [51,52,53].

*KEAP1* loss-of-function (LOF) and *NFE2L2* gain-of-function (GOF) mutations exhibited a mutually exclusive pattern, demonstrating that the overexpression of NRF2 may contribute as pivotal mechanism in the selection of specific cancers or stages of tumor progression, including lung cancer [54]. A total of 174 somatic mutations in *KEAP1* have been found, predominantly clustering in the intervening region (IVR) domain with missense/pathogenic significance (e.g., p.D236H, p.E242K, p.S243C, p.R272C, p.D294Y) [55]. Chemical modifications of cysteines within these domains disrupted the NRF2-KEAP1 interactions [17]. Meanwhile, *KEAP1* gene mutations at KELCH1-6 domains (e.g., p.R320Q, p.G364C, p.L413R, p.A466P, p.W544C, p.G570V), which interact with N-terminal Neh2 domain of NRF2, resulted in a dysregulated ability of KEAP1 to repress NRF2 [56,57,58,59].

Additionally, *KEAP1* heterozygous mutations contribute to NRF2 hyperactivation, as the loss of both *KEAP1* gene copies is not a critical event [60]. This can be explained by mutations in the Kelch-like domain acting with a dominant negative effect by forming heterodimers with wild-type proteins and disrupting NRF2 association [61]. On the other hand, somatic mutations in *NFE2L2* gene were found to be mainly located in the protein domains of DLG or ETGE (e.g., p.D29N, p.R34G, p.E79K, p.G81V). Aminoacidic NRF2 changes inhibited *KEAP1*-mediated NRF2 degradation, leading to its stabilization, abnormal nuclear accumulation with constitutive activation of cytoprotective enzymes [62].

Interestingly, most mutations that occurred in *KEAP1* or *NFE2L2* genes aligned with a smoking-associated mutational signature that predominates in early tumorigenesis, although only a very small subset showed a tendency to co-occur [63]. In LUSC, these genetic alterations recurred in about a third of all diagnosed cases, more frequently these are mutational events combined with copy number variations (CNVs) [51]. Over the years, in a pulmonary large-cell neuroendocrine carcinoma (LCNEC) cohort, Fernandez-Cuesta and colleagues identified *KEAP1* genetic alterations as a specific molecular hallmark with adenocarcinoma-like features [64].

*KEAP1* mutations frequently coexist with other genetic alterations, suggesting cooperative events that drive their selection [65]. For example, it has been reported that *KEAP1* alterations often co-occur with *STK11* (serine/threonine kinase 11) and *KRAS* mutations in LUAD; likewise, *NFE2L2* and *TP53* co-mutations but in lung squamous cell carcinoma (LUSC) [50]. *KRAS-mutant* lung adenocarcinomas often carried loss-of-function mutations in *KEAP1* [66], and co-mutations involving the PI3K-AKT-mTOR and NRF2 pathways, such as *NFE2L2* amplification combined with loss of *STK11*, have also been observed [67].

Tao and collaborators were among the first to offer compelling evidences that oncogenic *KRAS* upregulates NRF2 transcription levels via 12-O-tetradecanoylphorphorbol-13-acetate (TPA) at the exon 1 within its promoter region. In a murine model, they demonstrated that the inhibition of NRF2 pathway effectively contributed to overcome *KRAS*-mediated chemoresistance [68]. Similarly, in the context of resistance to targeted therapy, *NFE2L2* mutations may enhance cell survival under crizotinib treatment, enabling the acquisition of additional resistance mutations over time [69]. Arbour and collaborators identified a molecular subtype of *KRAS-mutant* NSCLC with co-mutations in *KEAP1* and *NFE2L2* genes, where patients had significantly shorter overall survival (OS), and a reduced duration of platinum-based chemotherapy response compared to other *KRAS-mutant* NSCLC patients [70].

These findings supported previous preclinical studies highlighting the role of NRF2-KEAP1 pathway deregulation in increasing resistance to radiation therapy, immunotherapy, and chemotherapy agents [71,72]. In this context, *KEAP1-driven* co-mutations were also found unresponsive to immunotherapy, despite high tumor mutational burden (TMB), with a decreased OS in LUAD patients [73]. In this subtype of lung cancer, Zavitsanou and coworkers demonstrated that *KEAP1-mutant* tumors were able to suppress dendritic cell and T cell responses, creating an immunosuppressive phenotype that could drive resistance to immunotherapy. Using Clustered Regularly Interspaced Short Palindromic Repeats—CRISPR associated protein 9 (CRISPR-Cas9) editing revealed that NRF2 pathway hyperactivation might contribute to impaired immune responses in *KEAP1-mutant* tumors in LUAD antigenic model and LUAD patient samples [74].

Given the significant impact of NRF2-KEAP1 genetic alterations on treatment resistance and patient outcome, the modulation of this pathway represents a promising therapeutic strategy [75]. Goeman et al. defined a distinct molecular subtype of chemotherapy-resistant and shorter OS and progression-free survival (PFS) in LUAD patients based on *NFE2L2* and *KEAP1* mutations profiling. Additionally, this study reported that *KEAP1* mutations were enriched in patients with high TMB but were associated with low T-cell infiltration, suggesting a key role for *KEAP1* alterations in immune evasion [76].

Similarly, *KEAP1* mutations were found to be associated with poorer clinical outcomes in LUAD patients receiving pembrolizumab in combination with carboplatin and pemetrexed, the standard first-line treatment options for driver-negative non-squamous NSCLC [77]. As a result, these findings suggested an enhanced NRF2 activity and functional inactivation of *KEAP1* in order to discriminate a molecular signature of specific subtype of NSCLC, defined by resistance to treatment and accelerated disease progression [78].

Beyond genetic mutations, the NRF2-KEAP1 pathway is also controlled by epigenetic mechanisms, including DNA methylation, histone modifications, miRNAs and lncRNAs expression, which play a pivotal role in the regulation of gene expression without altering DNA sequence [56]. Ongoing research in this field aims to develop interventions targeting *NRF2-KEAP1* epigenetic modifications that can be reversible or inherited during cell division, with the potential for chemoprevention, overcoming resistance and improving prognosis for NSCLC patients [79].

Recently, Elshaer and colleagues reported that DNA methylation alterations were associated with *KEAP1* mutations in LUAD patients, potentially shaping the NRF2 regulatory network functions. Using the Illumina Infinium Human DNA Methylation 450 K array platform, they demonstrated that the 8-gene signature of NRF2 targets exhibited low cytosine-guanine (CpG) dinucleotides methylation and increased gene expression levels in patients harboring *KEAP1* mutations [80].

DNA methylation of *KEAP1* has been more frequently studied than *NFE2L2* across multiple cancer models, revealing distinct methylation patterns at *KEAP1* promoter region when comparing normal and tumor lung tissues [81,82,83]. In vitro, Muscarella et al. reported that approximately 50% of NSCLC and 42% of SCLC cell lines showed a global DNA methylation at the *KEAP1* promoter, thus reducing gene expression. Moreover, an epigenetic screening of 47 NSCLC patient samples revealed *KEAP1* methylation in 47% of cases by quantitative methylation-specific PCR (qMSP) to detect a distinct group of CpG sites [84].

Methylation studies have revealed significant changes in KEAP1 epigenetic patterns in cancer cells from lung and other sides, mainly at CpG sites level at 5′ end, where the main *KEAP1* CpG island was located [85,86]. More specifically, the first seven single CpG sites (1–7, P1a region) that were found to be significantly more methylated compared to the last six CpG sites (8–13, P1b region) at the P1 promoter region of lung cancer cells [87]. Previous studies have validated the binding of transcription factors, Specificity protein (Sp-1) and Activator Protein 2 (AP-2) to the *KEAP1* promoter region at P1a region. As consequence, in lung cancer cells this binding is inhibited due to hypermethylation, suggesting the epigenetic interference in the modulation of KEAP1 expression in cancer cells [88]. Our group recently developed *OPERA_MET-A*, a multigene Next Generation Sequencing (NGS) panel, designed to detect differentially methylated cytosines across multiple solid cancer-related genes with notable results [89]. The NGS analysis confirms that the CpG methylation patterns of *KEAP1* promoter can occur symmetrically in double-stranded, thus corroborating the effect of hypermethylation in suppressing KEAP1 gene expression and function [89]. Moreover, since aberrant methylation is reversible through epigenetic drugs, treatment of lung cancer cell lines, including lung carcinoid, small cell lung cancer (SCLC), and adenosquamous carcinoma (ASC), with the DNA methylation inhibitor 5′-aza-2′-deoxycytidine (decitabine; DAC) can restore KEAP1 expression by demethylating its promoter P1 region. Additionally, an inverse correlation was observed between KEAP1 expression and promoter methylation following DAC treatment [87].

Another pharmacological approach using DNA methyltransferase (DNMT) inhibitor genistein has been shown to induce demethylation of *KEAP1* CpG promoter islands, leading to increased transcript levels with an overexpression of NRF2, GSS (Glutathione synthetase), and HO-1 transcription levels [90]. Moreover, epigenetic regulation of *NRF2* is influenced by extra-terminal (BET) proteins, which interact with acetylated lysine residues on histones and non-histone proteins to either activate or repress NRF2-dependent genes [91]. Li and colleagues focused on the role of histone modification that regulates *NFE2L2* repression through EZH2, a component of the polycomb PRC2 complex. EZH2 has been shown to inhibit lung cancer cell proliferation by silencing *NFE2L2* expression, which led to an increased trimethylation of histone H3 lysine 27 (H3K27me3) marks at its promoter [92].

Murray-Stewart and collaborators demonstrated that histone deacetylation could reactivate another epigenetic alteration in NRF2-KEAP1 signaling pathway. This involves miR-200a, which has been shown to destabilize KEAP1 mRNA levels in lung tumor cell lines resistant to polyamine analogs [93]. NRF2-dependent regulation of miR-1 and miR-206 plays a crucial role in NSCLC proliferation and tumorigenesis by modulating the pentose phosphate pathway (PPP). In primary tumor samples, these miRNAs were inversely correlated with PPP gene expression, while elevated expression of NRF2 target genes was associated with poor prognosis [94].

The overexpression of miR-155 mimic, designed to restore the endogenous miRNA activity, enhanced colony formation and cell migration while leading to a reduction in apoptosis in the human lung ASC resistant A549 (A549R) cell line [95]. This effect was significantly associated with high NRF2, NAD(P)H Quinone Dehydrogenase 1 (NQO1) and HO-1 transcription levels. Conversely, the inhibition of miR-155 expression strongly reduced NRF2, NQO1, and HO-1 expression, accompanied with a decreasing in B cell lymphoma gene 2 (BCL2)/BCL-2-associated protein X (BAX) ratio. Finally, these results suggested that miR-155 mediated chemotherapy resistance by activating NRF2 signaling pathway with an inhibition of cellular apoptotic process [95].

Similarly, Duan and coworkers demonstrated that miR-421 overexpression in A549 lung cancer cells directly targeted 3′-untranslated region (3′UTR) of *KEAP1* gene, leading to its downregulation with an increased proliferation and invasion but decreasing apoptosis process [96]. In addition, Yin and colleagues investigated the role of miR-144-3p in drug resistance in a set of lung cancer cells [97]. In this context, the expression of miR-144-3p was activated by NRF2 upon the binding to AREs in its promoter region. The targeting of this miRNA led to the downregulation of NRF2, resulting in decreased gene expression levels that were found to be associated with cisplatin resistance [97].

Furthermore, preliminary data were reported on miRNAs interacting with *NRF2* and *KEAP1* by gene expression profiling [98]. Our group demonstrated that the downregulation of miR-27 family (miR-27a and miR-27b) and the upregulation of miR-200 family (miR-200a), could significantly modulate NRF2-KEAP1 activity in NSCLC and SCLC cell lines panels, as well as in a training set of 29 tumors and adjacent normal tissues from NSCLC patients [99].

Interesting findings came from smoke and cancer-associated lncRNA 1 (SCAL1) and Nuclear LUCAT1 (NLUCAT1) in lung cancer cells through the regulation of oxidative stress. SCAL1 is considered the first NRF2-activated lncRNA, which is identified in airway epithelial cells against oxidative stress [100]. It has been reported that there are increased levels of SCAL1 upon cigarette smoking, with a significant correlation with *NFE2L2* mutations [101]. Recently, Moreno Leon et al. reported that the inhibition of NF-κB-dependent transcription decreased the hypoxia-dependent expression of NLUCAT1, an NRF2-induced lncRNA [102]. Using *CRISPR-Cas9*, they demonstrated that *NLUCAT1* knockdown in lung adenocarcinoma cells caused a lower proliferation rates, while an increasing ROS levels and sensitivity to cisplatin-induced apoptosis was found. These evidences suggested that this lncRNA is involved in the regulation of the NRF2-mediated antioxidant response with an impact on cisplatin resistance in lung cancer cells [103].

A summary of genetic, epigenetic, and molecular alterations of the NRF2-KEAP1 pathway in lung cancer and their clinical significance is shown in Table 1.

## 4. NOTCH Signaling Hub in Lung Cancer: A Central Regulator of Tumor Progression

Dysregulation of NRF2 and NOTCH transcription factors is frequently observed in tumorigenesis, as their related-aberrant signaling can lead to uncontrolled proliferation, impaired differentiation, and resistance to apoptosis [104]. Among these, the NOTCH pathway stands out as a critical regulator of cellular processes linked to both normal development and cancer progression [105]. NOTCH signaling plays a pivotal role in maintaining stemness and orchestrating organogenesis, requiring precise regulatory control [106]. While proper NOTCH activity ensures orderly cellular differentiation, its dysregulation is frequently linked to carcinogenesis [107].

NOTCH plays a context-dependent role in cancer, acting as an oncogene in several malignancies via hyperactivation [108], but also as a tumor suppressor in specific settings [109]. It is fundamental in the regulation of differentiation and tissue repair following injury [2], and it is essential for developmental processes across various organs including skeletal, hepatic, cardiac, and hematopoietic systems. A hallmark of NOTCH-mediated development is its temporal coordination of control overgrowth and differentiation, contributing it to promote or inhibit proliferation based on context [110]. In cancer, deregulated NOTCH signaling leads to epithelial–mesenchymal transition (EMT) [111], cancer stem-like cell maintenance [106], angiogenesis [112], and modulation of TME through immune cell recruitment and stromal interactions [113].

**Figure 2:**
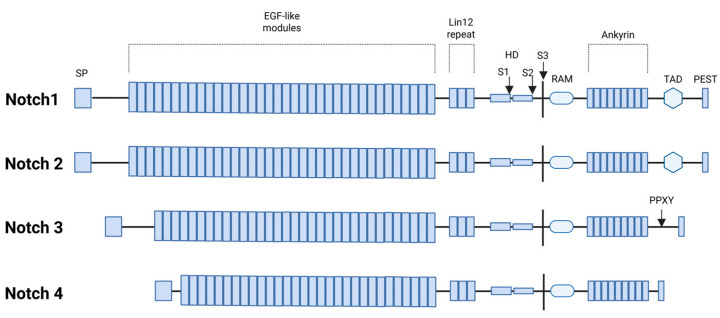
Functional domains of human NOTCH1-4 proteins: signal peptide (SP), epidermal growth factor-like repeats (EGF), LIN-12 repeat (LNR) as receptors for intercellular signals, heterodimerization domain (HD), S1/S2/S3 proteolytic cleavage sites, RBP-Jkappa-associated module (RAM), transcriptional activation domain (TAD), and the polypeptide sequence rich in proline (P), glutamic acid (E), serine (S), and threonine (T) residues. Additionally, the NOTCH3 receptor contains a proline-rich Pro-Pro-x-Tyr (PPXY) motif that serves as a recognition site for the WW domain-containing protein 2 (WWP2), an endocytic regulatory protein (Created with BioRender.com, Licensing and Agreement number RY285EXIU5, accessed on 15 April 2025).

Like other receptor-mediated signaling pathways, NOTCH network comprises three key components: receptors, ligands, and downstream effectors [2]. In mammals, there are four NOTCH receptors (NOTCH1, NOTCH2, NOTCH3, and NOTCH4, which are transmembrane proteins consisting of three main segments: NOTCH extracellular domain (NECD), that mediates ligand binding; a transmembrane domain (TMD), that anchors the receptor in the membrane; a NOTCH intracellular domain (NICD), that functions as the active signaling component upon cleavage (Figure 2). Five ligands interact with the extracellular domain of NOTCH receptors, classified into two groups based on their structural features: Serrate-like ligands: Jagged1 (JAG1) and Jagged2 (JAG2), which contain a cysteine-rich domain; delta-like ligands: DLL1, DLL3, and DLL4, which lack the cysteine-rich domain [114,115].

Following ligand–receptor interaction, a cascade of proteolytic cleavages releases the NICD, which translocate into the nucleus and interact with co-activator and co-repressor complexes to regulate the transcription of target genes [116]. These downstream effects shape diverse cellular outcomes, underscoring the pathway’s critical role in normal development as well as in oncogenic transformation.

Going into detail, we can distinguish between a canonical and non-canonical NOTCH signaling pathway [117]. In the canonical pathway, the NICD is released following metalloprotease-mediated proteolytic cleavage. The final cleavage is performed by γ-secretase, enabling NCID to translocate into the nucleus, where it binds to specific DNA sequences to promote gene transcription [118]. Proteolytic cleavage can also occur at the endosomal level during vesicular trafficking processes, adding another layer of regulation [119].

In the canonical pathway, NOTCH likely interacts with complex transcriptional machinery, led by the CBF-1/suppressor of hairless/Lag1 (CSL) complex [120]. CSL recruits various co-transcription factors that modulate gene expression, ensuring precise control of developmental and cellular processes [121].

However, NOTCH can also signal through non-canonical pathways, interacting with key molecular regulators such as NF-κB, mTOR, PTEN, AKT, wingless-type MMTV integration site family (Wnt), and Hippo signaling [2]. Non-canonical ligands lack the essential Delta/Serrate/Lag-2 (DSL) domain required for interaction with NOTCH [122]. These ligands include a structurally diverse group of proteins, including integral membrane proteins and glycosylphosphatidylinositol (GPI)-linked membrane proteins [123]. Although non-canonical ligands may have important biological functions, their roles have been insufficiently explored in vivo, and their impact on organ development and disease progression remains not fully understood.

Moreover, NOTCH signaling plays a crucial role in sustaining tumor growth under hypoxic conditions, thereby reducing the efficacy of pharmacological and radiotherapeutic interventions [124]. This ability to support cell survival in low-oxygen environments underscores the role of NOTCH as a key factor in tumor aggressiveness and therapy resistance [125]. The four isoforms of the NOTCH receptor have been found both overexpressed and mutated in patients with lung cancer. Gene sequencing analyses have revealed *NOTCH* gene mutations in various human cancers, particularly in both NSCLC and SCLC [126,127]. These mutations contribute to tumor initiation and progression by promoting stem cell self-renewal, enhancing cellular plasticity, and fostering resistance to therapy [128].

The role of NOTCH family protein as oncogenes or tumor suppressors is complex; NOTCH1 has also been shown to be a tumor suppressor and overexpressed in association with lymph node metastases in patients with NSCLC [126]. NOTCH1 has been much more associated with stem-like characteristics and self-renewal capacity of NSCLC cells; NOTCH2 in mitogen-activated protein kinase (MAPK) pathways with growth and development of NSCLC; NOTCH3 has been linked to TGF-β-mediated EMT processes via transcriptional repression of Zinc finger E-box binding protein 1 (ZEB1), NOTCH4 has also been associated with anti-tumor immunity, promoting the infiltration of diverse immune cells populations [129].

NOTCH involvement in EMT has been associated with reduced E-cadherin (E-cadherin) and Snail expression leading to destabilization of the epithelial structure in an intricate signaling network mediated by TGF-β; hyper-activation of cyclins D1 was also associated with EMT in NOTCH signaling [130]. From TCGA data and global analysis of expression profiles, it has been reported that in both LUSC and LUAD opposing roles of NOTCH1 has been revealed, being a suppressor in LUAD and an oncogene in LUSC [105]. High levels of Jag1, DLL1, NOTCH1, and NOTCH2 mRNA were associated with better OS in lung adenocarcinoma patients, while Jag2, DLL3, and NOTCH3 mRNA correlated with poor survival [131].

The opposing prognostic roles of Jag1 and Jag2 were attributed to their mutual inhibition, as Jag1 and Jag2 levels were inversely regulated [132]. NOTCH2 expression was lower in NSCLC patients compared to healthy lung tissue [133]. Additionally, NOTCH3 gene polymorphisms (G684A and C381T) were linked to increased lung cancer susceptibility [134]. Finally, ligands, such as DLL3, play a crucial role in the differentiation plasticity of SCLC tumor cells, influencing cell proliferation, EMT, chemotherapy resistance and the expression of immune biomarkers. DLL3, widely expressed in SCLC tumor cells, represents a promising therapeutic target [135].

Given the pleiotropic nature of NOTCH signaling, therapeutic strategies targeting this pathway must carefully balance its dual role in tumorigenesis. While NOTCH inhibitors show promise in treating specific cancers, they must be precisely tailored to avoid disrupting their essential physiological functions involved in tissue homeostasis and regeneration [136].

**Figure 3:**
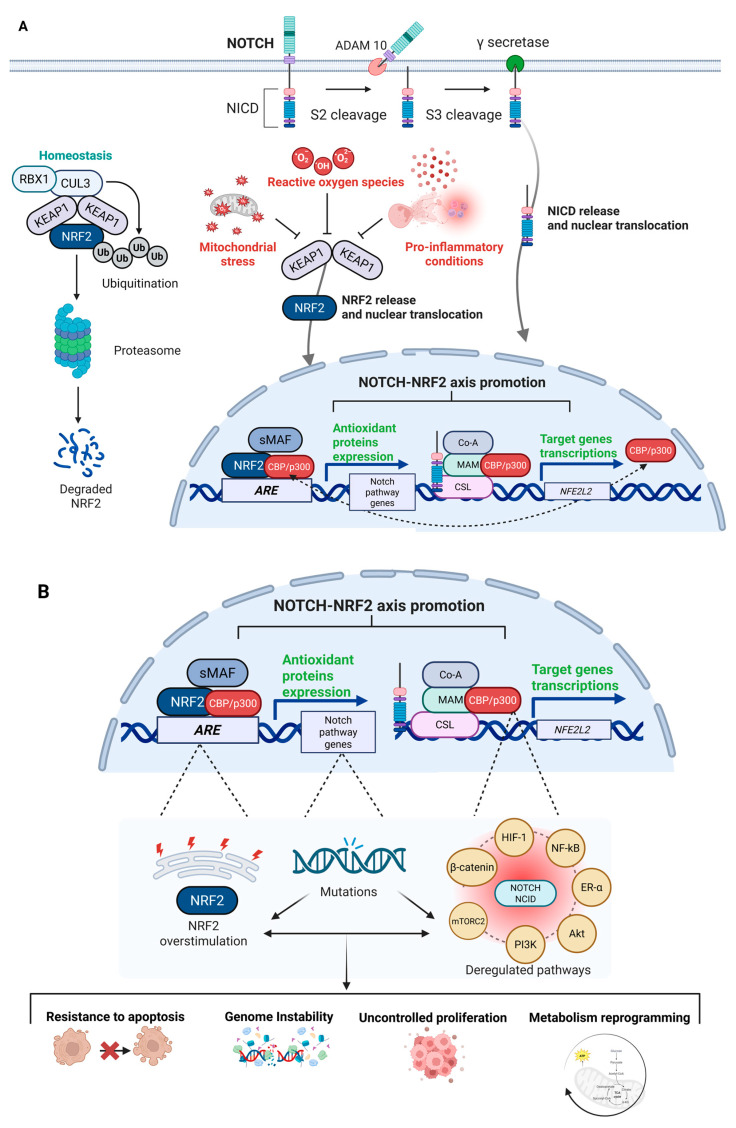
Physiopathological NRF2–NOTCH axis overview. General overview of the NRF2–NOTCH axis, highlighting the main factors and protein complexes involved in the activation of both pathways. (**A**) Under homeostatic conditions, KEAP1 directs NRF2 towards ubiquitination and subsequent degradation via the proteasome complex. During stress conditions—often linked to altered energy metabolism, mitochondrial dysfunction, and accumulation of ROS, which amplify pro-inflammatory signals—KEAP1 undergoes conformational changes that allow NRF2 to translocate into the nucleus, where it functions as a transcription factor by binding to AREs to initiate a cytoprotective response. Similarly, under physiological conditions, the NOTCH pathway regulates normal development and cell differentiation, thereby contributing to cellular homeostasis. Upon ligand binding, the NOTCH receptor undergoes a series of proteolytic cleavages, culminating in cleavage by γ-secretase, which releases the NICD. NICD then translocates to the nucleus, where it acts as a transcriptional regulator of specific target genes. NICD can interact with the promoter of the *NFE2L2* gene to stimulate NRF2 transcription, or it can cooperate with transcriptional co-activators such as CBP/p300 to enhance the transcriptional activity of NRF2 on its target genes. Conversely, NRF2 can upregulate components of the NOTCH pathway by binding to AREs located in the promoters of various genes, including those involved in NOTCH signaling, thereby promoting their expression. Beyond these direct mechanisms, the interconnection between the NOTCH and NRF2 pathways is part of a much broader and more intricate signaling network, with multiple intermediate pathways activated by both axes (Created with BioRender.com, Licensing and Agreement number YR285EY4B1, accessed on 15 April 2025). (**B**) Mutations or dysregulation within the NRF2–NOTCH axis can lead to aberrant and dysfunctional activation. In cancer, hyperactivation of NRF2—often triggered by persistent stress signals—can be exploited by tumor cells to survive hostile conditions and may in turn contribute to increased NOTCH signaling. Likewise, deregulated NOTCH activity can either upregulate or fail to appropriately activate NRF2. Beyond their oncogenic potential, the deregulation of NOTCH and NRF2 activities can affect a multitude of downstream effectors, such as HIF-1, NF-κB, ER-α, AKT, PI3K, mTORC2, and β-catenin. The deregulation of these interconnected pathways facilitates the acquisition of a set of cancer hallmarks as well as resistance to apoptosis, genomic instability, uncontrolled proliferation, and metabolic reprogramming. The biological complexity of NRF2–NOTCH axis defines its ability to rewire multiple tumor-promoting networks, fueling cancer development and progression (Created with BioRender.com, Licensing and Agreement number ST285EYE6Y, accessed on 15 April 2025). Abbreviations: KEAP1, Kelch-like ECH-associated protein 1; NRF2, Nuclear factor erythroid 2-related factor 2; Cul3, Cullin 3; RBX1, RING-box protein 1; Ub, Ubiquitin; NOTCH, Neurogenic Locus NOTCH Homolog; ADAM10, A Disintegrin And Metalloprotease 10; NICD, NOTCH Intracellular Domain; sMAF, Small Musculoaponeurotic Fibrosarcoma oncogene homologs; CBP/p300, CREB-binding protein/E1A-binding protein p300; ARE, Antioxidant Response Element; Co-A, Co-Activator; MAM, Mastermind-like protein; CSL, CBF-1/suppressor of hairless/Lag1; ROS, Reactive Oxygen Species; HIF-1, Hypoxia-Inducible Factor 1; NF-κB, Nuclear Factor kappa-light-chain-enhancer of activated B cells; ER-α, Estrogen Receptor alpha; Akt, Protein Kinase B; PI3K, Phosphoinositide 3-Kinase; mTORC2, Mechanistic Target of Rapamycin Complex 2; β-Catenin, Beta-Catenin.

The physiopathological overview of the NRF2–NOTCH axis and their crosstalk is illustrated in Figure 3A, while Figure 3B depicts the potential consequences of dysfunctional activation.

## 5. Genetic and Epigenetics Hallmarks of NOTCH in Lung Cancer

The alterations characterizing NOTCH signaling during lung cancer pathogenesis might be finely tuned either by somatic mutations and epigenetic modifications, acting as a double-edge sword for its progression [137]. In a recent study, a targeted deep sequencing was conducted assessing 48 tumor-related genes on 153 samples from 55 LUAD and LUSC patients using different sources: formalin-fixed paraffin-embedded (FFPE) tumor tissues, pleural effusion supernatant, pleural effusion cell sediments, white blood cells, oral epithelial cells, and plasma [138].

Mutations were detected in 96% of patients and in 83% of the selected genes, therefore exhibiting a characteristic mutational pattern. Among them, *NOTCH1* was one of the top eight mutated genes (36.4%) making it the second most frequently altered gene after *TP53* [138]. Notably, 70% of plasma samples harbored *NOTCH1* mutations, a frequency significantly higher than the 20% observed in tumor tissues. Furthermore, copy number loss of *NOTCH1* was detected in 21% of patients, underscoring the gene’s susceptibility to both sequence-level mutations and structural loss [138]. These mutations included a heterogeneous group of missense mutations: synonymous mutations, and nonsense (truncating) mutations. Many of these mutations clustered within functional domains such as the calcium-binding epidermal growth factor-like repeats (EGF) domain, the EGF-like domain, the (Lin-12/NOTCH repeat (LNR) domain, the Ankyrin repeats, and the proline (P), glutamic acid (E), serine (S), and threonine (T) as well as PEST domain. Notably, the presence of synonymous mutations, which can influence splicing or gene regulation, suggesting potential regulatory roles beyond direct protein alteration [138].

Among these mechanisms, Westhoff et al. demonstrated that NOTCH1 expression in lung cancer relies on NUMB Endocytic Adaptor Protein (NUMB) activity and somatic mutations acquisition. As contextual evidence, they demonstrated an inverse correlation between Numb, regulated post-translationally in lung cancer, and NOTCH1 accumulation, supported by the expression analysis of the *NOTCH*-target gene HES family bHLH transcription factor 1 (HES1). Despite the significative inverse correlation, the authors were able to identify an increased expression of HES1 along with a sustained expression of NUMB, hinting towards a possible gain-of-function *NOTCH1* mutations [139]. Corroborating this scenario, they identified four different heterozygous mutations by sequencing the entire C-terminal region of the *NOTCH1* on the 49 NSCLC cohort, eventually also showing predictive features for a poor prognosis in the *TP53*-mutated subgroup [139].

In line with this, Huang et al. recently assessed *NOTCH1* mutations in 55 out of 963 patients with NSCLC, accounting for 5.7% of cases; a similar percentage to the one they found by further analyzing two public available databases [140]. Of note, mutations were more common in male patients 65 years old at least, smokers and patients with squamous-cell carcinoma. An independent work showed that SNPs affected both *NOTCH1* and *NOTCH2* genes in lung biopsies obtained from smoker patients [141].

Interestingly, the median of tumor mutational burden for *NOTCH1* mutated tumors was significantly higher than for *NOTCH1* wild-type tumors, because of the alterations in DNA damage and repair-related genes. Among the most affected pathways, the PI3K-AKT-mTOR signaling was overactivated in this context [142]. Therefore, Programmed Cell Death Ligand 1 (PD-L1) expression in *NOTCH1* mutated tumors was significantly higher than in *NOTCH1* wild-type tumors, therefore enriching the milieu in CD8+ T cells, hallmarks of a highly inflammatory tumor microenvironment [140]. *NOTCH1*, however, is not the only gene mutated from the NOTCH family. It has been reported indeed that a chromosomal translocation involving t(15;19) regulates NOTCH3 expression in 40% of lung cases, leading to the overexpression of a full-length functional protein [143].

Besides the plethora of mutations that have been reported affecting NOTCH members, a further regulation level relies on the epigenetic control of this family of genes. However, only a few evidences are pointing to the epigenetics control sitting upstream NOTCH regulation. The assessment of NUMB expression in public array databases revealed a marked decrease in its expression of lung cancer. These data correlate with a significant hypermethylation of its promoter, as confirmed by assessing this epigenetic modification of tumor biopsies in comparison to healthy tissues [144]. Consequently, NOTCH cascade is activated, eventually promoting cancer stem cell-like properties and resistance to chemotherapy in NSCLC cell lines.

There is an increasing need to overcome NOTCH expression to counteract lung cancer progression [127]. In this context, it has been shown that Sirtuin proteins, nicotinamide adenine dinucleotide (NAD)-dependent deacetylases and ADP-ribosyl transferases (class III histone deacetylase enzymes, HDAC), are involved in regulating NOTCH1 expression via DNMT1. Subramani et al. showed that *SIRT6* silencing might represent a strategy for mitigating lung cancer progression; upon its knockdown DNMT1 is acetylated and stabilized, therefore promoting *NOTCH1* promoter methylation, and thus repressing its expression [145].

On this basis, Su et al. recently reported that DNMTs activity in NSCLC cell lines can also be modulated by Evodiamine, an alkaloid derived from Euodiae Fructus [146]. The extract exerts its activity by reducing cell viability in vitro and in vivo in rodent models. Furthermore, this alkaloid induced G2/M cell cycle arrest, inhibited cell migration and reduced stemness in cultured NSCLC cells. These outcomes appear to be closely linked to increased NOTCH3 methylation, which occurs following treatment with the extract due to the stabilization of DNMTs [146].

More recently reported epigenetic modification affecting NOTCH family members’ expression involves the non-coding RNAs. It has been reported that miR-34a inhibited the NOTCH signaling pathway by reducing the expression of HES-1, NOTCH1, and Survivin, leading to decreased cell growth and invasiveness and promoting apoptosis in NSCLC cells [147]. Xue et al. also explained the contribution of miR-200 in this context. They reported that miR-200 mediated the interaction between LUAD cells and neighboring cancer-associated fibroblasts (CAFs) by targeting the NOTCH ligands Jagged1 and Jagged2, which in turn activated the NOTCH signaling pathway in CAFs and ultimately inhibited lung ASC metastasis [148].

Similarly, cells stably overexpressing lncRNA-p21 and upregulated Jagged1 promote NOTCH1 activation, thus suggesting another mechanism by which lncRNAs modulate NOTCH cascade. In addition to this, lncRNA AGAP2 Antisense RNA 1 (AGAP2-AS1), it has been described to activate the NOTCH signaling pathway in lung cancer cells by upregulating NOTCH2 through the sequestration of miR-296, ultimately promoting malignant cellular behaviors [149]. Likewise, lncRNA Small Nucleolar RNA Host Gene 11 (SNHG11) enhanced NOTCH3 expression sponging miR-193a-5p, thereby activating the NOTCH signaling pathway and facilitating the progression of lung adenocarcinoma. The circ_0000190 was shown to reverse luteolin-induced suppression of lung cancer progression by activating the NOTCH1 signaling pathway sponging miR-130a-3p [150,151]. A recent scientific report also highlighted the significative overexpression of lncRNA plasmacytoma variant translocation 1 (PVT1) in NSCLC tissues and cell lines, where it promoted NSCLC cell proliferation, migration, and invasion. Corroborating this, silencing of *PVT1* suppressed the expression of Yes-associated protein 1 (YAP1) and inhibited the activation of the NOTCH1 signaling pathway. The mechanism involves EZH2-mediated methylation of the miR-497 promoter, which suppresses miR-497 transcription and results in the upregulation of its target, YAP1. This cascade ultimately activates the NOTCH signaling pathway, thereby promoting epithelial–mesenchymal transition (EMT), invasion, and metastasis [152].

Overall, all these findings highlight the intricate regulatory network governing NOTCH signaling in lung cancer, where genetic mutations and non-coding RNAs collectively shape tumor progression, microenvironment interactions, and therapeutic responses [153]. Table 2 summarizes the alterations targeting NOTCH signaling in lung cancer, highlighting genetic mutations, epigenetic modifications, miRNAs and non-coding RNAs and their impact on clinical outcomes.

## 6. Modulation of NRF2–NOTCH Signaling Across Lung Cancer Subtypes and Treatment Responses

The interaction between NRF2 and NOTCH signaling pathways is increasingly recognized as context-dependent, with variable effects across different lung cancer subtypes and under distinct therapeutic pressures [11]. Genetic analyses have shown that the mutational landscape of *NFE2L2* differs significantly between LUAD and LUSC [154]. Specifically, *KEAP1* mutations are more commonly observed in LUAD, whereas *NFE2L2* mutations predominantly occur in LUSC [72].

In LUAD, mutations in *KEAP1*, *STK11*, *NRF2*, and *SMARCA4*, which drive the redox-high phenotype, have been shown to be associated with a reduction in tissue-resident memory CD8⁺ T cells, thereby attenuating the efficacy of immune checkpoint inhibitors [155]. In this histology, activating mutations in *NFE2L2* or loss-of-function mutations in *KEAP1* lead to constitutive activation of NRF2, which has been linked to tumor progression and chemoresistance [71]. Furthermore, NRF2 activation can upregulate the components of the NOTCH pathway, promoting a stem-like phenotype that enhances resistance to chemotherapy and targeted therapies, like EGFR inhibitors (EGFRi) [7].

In LUSC, the *NFE2L2* mutations are also common and correlate with increased resistance to radiotherapy [156]. Interestingly, NRF2 activation in LUSC has been shown to suppress NOTCH signaling, which in turn disrupts differentiation pathways, leading to enhanced cell proliferation and survival [157]. Moreover, NRF2-driven chemoresistance in LUSC can also be attributed to its role in modulating the cellular response to cisplatin and other DNA-damaging drugs [158]. In a high-grade neuroendocrine (NE) cancer, like SCLC, where *KEAP1* mutations are less frequent, epigenetic silencing of *KEAP1* can represent a leading mechanism to aberrantly activate NRF2, contributing to chemoresistance, particularly to agents like etoposide and platinum-based therapies [83].

The interplay between NRF2 and NOTCH in SCLC is particularly notable, as NOTCH signaling is often suppressed, leading to the maintenance of the NE phenotype, which is typically associated with poor prognosis and therapy resistance [135]. Basically, on one hand, the inhibition of NRF2 activity has demonstrated potential in reversing chemoresistance in preclinical models; on the other, the modulation of NOTCH signaling is under investigation as a strategy to promote cellular differentiation and enhance tumor sensitivity to chemotherapy [159].

These findings suggest that NRF2 and NOTCH pathways cooperate in modulating tumor cell survival and therapeutic resistance, underscoring the need for novel therapeutic strategies that target both pathways simultaneously.

## 7. Clinically Relevant Mutations and Epigenetic Modifiers of the NRF2-KEAP1 and NOTCH Pathways in Lung Cancer

Several specific genetic and epigenetic alterations affecting the NRF2 and NOTCH pathways have been identified in many clinical cohorts of lung cancer patients, and they hold significant potential for improving patients’ stratification and guiding therapeutic decision [7]. Previous studies have demonstrated that the activation of the NRF2 pathway contributes to resistance against various anticancer therapies, with early-stage NSCLC patients harboring *KEAP1* or *NFE2L2* mutations exhibiting a higher risk of local recurrence following radiotherapy [160,161]. Furthermore, recent studies suggest that the activation of the NRF2-KEAP1 pathway correlates with poorer OS post-chemotherapy. In particular, patients with metastatic NSCLC harboring mutations in *KEAP1*, *NFE2L2*, or *CUL3* showed significantly shorter time to treatment failure and OS following first-line platinum doublet chemotherapy, compared to matched controls [71].

Analysis of data from the Memorial Sloan–Kettering Cancer Center (MSKCC) cohort demonstrated that *KEAP1* and/or *NFE2L2* genetic alterations could be significantly associated with increased TMB and elevated PD-L1 expression with a greater clinical benefit from immunotherapy than from other treatment modalities, suggesting potential predictive value of these mutations in guiding immunotherapeutic strategies [162]. Notably, mutations in *STK11* and *KEAP1* genes markedly influence the prognosis of patients with advanced NSCLC, especially when they co-occur with *KRAS* mutations, offering substantial potential to improve patient stratification for immunotherapy and thereby enhance clinical outcomes [163]. On the other side, several mutational landscape of the *NOTCH* gene family (particularly *NOTCH1* and *NOTCH3*), observed in a subset of SCLC, supports a context-dependent tumor-suppressive role. This underscores the importance of future research to investigate the clinically actionable impact of *NOTCH* loss, especially concerning treatment resistance and disease relapses in plasma cell-free DNA (cfDNA) [164].

On the epigenetic front, in molecularly stratified subpopulations of NSCLC, particularly in LUAD and LUSC cases lacking *KRAS* mutations, our group demonstrated a strong positive correlation has been observed between *KEAP1* promoter hypermethylation and elevated NRF2 mRNA expression, along with increased expression of multiple ARE-driven target genes. Notably, the epigenetic silencing of *KEAP1* was specifically linked to *KRAS* wild-type status in both LUAD and LUSC, being present in the *KRAS* wild-type subpopulations and absent in patients with *KRAS* mutations [165].

As expected, the hyperactivation of NRF2 leads to resistance across a wide spectrum of conventional and novel treatments, including chemotherapy, radiotherapy, and immunotherapy [166]. More recently, NRF2-KEAP1 pathway alterations have been associated with resistance to KRAS G12C inhibitor therapies [167], highlighting their potential utility in identifying patients who may benefit from NRF2-targeted inhibitors or redox-based combination treatments. Stratifying patients based on *KEAP1* or *NFE2L2* mutational status is increasingly being utilized to guide treatment decisions, including the exclusion of patients from ICI monotherapy trials due to their poor predicted response [168].

Moreover, integrated biomarker profiling that includes *KEAP1/NFE2L2*, *NOTCH*, and *KRAS/STK11* alterations may improve the identification of molecular subtypes associated with therapeutic resistance and worse clinical outcomes.

## 8. Metabolic Roles of NRF2 and NOTCH in Lung Cancer

Emerging evidence highlights the critical interplay between the NRF2 and NOTCH signaling pathways in lung cancer, where they also cooperate to reprogram metabolism in support of tumor growth, redox homeostasis, and therapy resistance [169,170,171]. This metabolic reprogramming enables cancer cells to meet increased energetic and biosynthetic demands through enhanced glycolysis, glutaminolysis, lipid catabolism, and antioxidant defense [172]. NOTCH signaling contributes by upregulating MYC, a master regulator of metabolism, in a genotype- and tissue-specific manner [173], while NRF2 regulates ROS homeostasis via induction of enzymes like NQO1, glutamate-cysteine ligase modifier subunit (GCLM), and heme oxygenase 1 (HMOX1) [174,175,176].

*NRF2* knockdown in NSCLC cell lines dramatically increases endogenous ROS levels and enhances sensitivity to radiation therapy [177]. Moreover, NRF2-mediated ROS homeostasis contributes to chemoresistance through the PI3K/Akt pathway [178]. The regulation of ROS by NRF2 has far-reaching metabolic consequences. It enhances nicotinamide adenine dinucleotide phosphate (NADPH) production by upregulating key enzymes such as glucose-6-phosphate dehydrogenase (G6PD), isocitrate dehydrogenase 1 (IDH1), malic enzyme 1 (ME1), and 6-phosphogluconate dehydrogenase (PGD), thereby supporting both redox homeostasis and biosynthetic pathways [94,178,179].

A recent work by Weiss-Sadan and colleagues has revealed that NRF2 activation can induce NADH-reductive stress, creating a metabolic vulnerability in lung cancer [180]. This study demonstrates that NRF2 activation, either through pharmacological inhibition of *KEAP1* or genetic mutations, leads to an imbalance in the NADH/NAD+ ratio, which disrupts mitochondrial function and sensitizes cancer cells to Complex I inhibition [180]. In NSCLC, NRF2 activation is associated with the overexpression of NADPH-producing enzymes and NADPH oxidases (NOX2 and NOX4), which together help maintain redox balance and fuel critical biosynthetic pathways essential for cancer cell growth [181,182].

In lung cancer cells, NRF2 activation correlates with increased lactate production and glucose uptake, conferring tumor aggressiveness in terms of invasion and metastasis [183]. Recent studies have identified in lung adenocarcinoma a metabolic phenotype (MP-III), characterized by high glycolytic activity and low lipid metabolism, associated with poor prognosis and metastatic potential [184]. This phenotype is driven by mutations in *TP53* and *KEAP1*, as well as deletions in *SETD2* (SET domain containing 2) and *PBRM1* (Polybromo 1) [184]. Notably, NRF2 hyperactivation in NSCLC is also linked to hypoxia-inducible factor 1α (HIF1α), further amplifying glycolytic activity by enhancing the expression of several key genes including hexokinase 2 (HK2), phosphofructokinase-2/fructose-2,6-bisphosphatase 3 (PFKFB3), pyruvate kinase isozymes M2 (PKM2) and lactate dehydrogenase A (LDHA) [185].

Concurrently, NOTCH-driven SCCs display elevated expression of glycolytic and serine biosynthesis enzymes, including aldolase A (ALDOA), glyceraldehyde 3-phosphate dehydrogenase (GAPDH), phosphoserine aminotransferase 1 (PSAT1), and serine hydroxymethyltransferase 2 (SHMT2), supporting nucleotide synthesis and antioxidant defense [186]. NOTCH signaling also promotes glutaminolysis by upregulating glutaminase (GLS), glutamic-oxaloacetic transaminase 2 (GOT2), and malate dehydrogenase 2 (MDH2), thereby fueling tricarboxylic acid (TCA) cycle intermediates and supporting reductive carboxylation [187].

These chemical reactions and pathways also involve glutathione metabolism. In fact, NRF2 controls NADPH synthesis by modulating the expression of enzymes that, such as ME1 and IDH1 [170,188]. NADPH provides the reducing equivalent necessary for the glutathione regeneration, via glutathione reductase (GR) and also serves as a cofactor for TRXR1 and NQO1, showing a clear correlation between the cellular redox status and metabolic functions regulated by NRF2. NRF2 enhances the synthesis of glutathione (GSH) by upregulating glutamate-cysteine ligase (GCL) and GSS, which helps counteract oxidative stress. Elevated GSH levels in LUSC contribute to resistance against radiation and chemotherapy [189,190].

NRF2 supports GSH metabolism by increasing cystine uptake via solute carrier family 7 member 11 (SLC7A11) and enhancing the expression of GCL and GSS, thereby strengthening antioxidant defenses and contributing to chemoresistance [188,189]. In addition, *NOTCH*-driven metabolic phenotypes include upregulation of nucleotide biosynthesis enzymes like nucleotide biosynthesis enzymes such as cytidine triphosphate synthase 1 (CTPS1) and guanosine monophosphate synthetase (GMPS), further linking metabolic reprogramming to therapy resistance [186].

In terms of lipid metabolism, NRF2 inhibits de novo lipogenesis via repression of stearoyl-CoA desaturase 1 (SCD1) and acetyl-CoA carboxylase 1 (ACC1), while promoting fatty acid oxidation (FAO) through the activation of genes such as carnitine palmitoyltransferase 1 (CPT1) and CD36, sustaining energy production under stress and minimizing lipid-induced ferroptosis [191]. These adaptations are accompanied by high expression of FAO genes like CPT1A, facilitating ROS clearance and tumor progression [192].

Among the numerous functions carried out by NRF2, its role as transcriptional activator of anti-ferroptosis genes is essential, as it prevents lipid peroxidation and the accumulation of free iron. In fact, the SLC7A11 cystine-glutamate transporter system and glutathione peroxidase 4 (GPX4) are two key regulators of ferroptosis process that are directly regulated upstream by NRF2 [193,194]. Recent studies have shown that cancer cells can evade ferroptosis induced by SLC7A11 or GPX4 inhibition through activation of the NRF2-ARE pathway, and that inhibiting NRF2 may help overcome this resistance to ferroptotic cell death [195,196].

NRF2 also modulates iron homeostasis by regulating ferritin heavy chain (FTH1) and HMOX1, which protect cells from ferroptosis [197]. Moreover, Mindbomb E3 Ubiquitin Protein Ligase 1 (MIB1), an E3 ubiquitin ligase, was able to stimulate NRF2 degradation in a NOTCH-independent manner, rendering cancer more responsive to ferroptosis inducers [198]. *MIB1* knockout induces NRF2 accumulation and resistance to ferroptosis, indicating that MIB1 may function as a positive regulator of ferroptosis through targeted NRF2 degradation [198]. In NSCLC, it has been documented that NRF2 activation stabilizes BTB and CNC Homology 1 (BACH1), a pro-metastatic transcription factor, by reducing free heme levels [183].

Finally, antioxidants like N-acetylcysteine and vitamin E can paradoxically promote lung cancer metastasis by reducing free heme levels and stabilizing BACH1, which activates glycolytic genes (*HK2* and *GAPDH*) to increase glucose uptake and lactate production [183]. Recent studies highlight the role of the Coenzyme Q (CoQ) and Ferroptosis Suppressor Protein 1 (FSP1) axis in mediating ferroptosis resistance in *KEAP1-mutant* tumors, presenting a promising novel therapeutic target [199].

The major metabolic pathways modulated by NRF2 and NOTCH signaling in the progression of lung cancer are reported in Table 3.

## 9. Targeting NRF2 and NOTCH Pathways in Lung Cancer: Current Drugs, Emerging Strategies, and Clinical Relevance

The therapeutic targeting of NRF2 and NOTCH pathways in lung cancer has emerged as a promising approach due to their critical roles in cancer cell survival, resistance to therapy, and tumor progression. Aberrant activation of NRF2 has been linked to increased tumorigenicity and unfavourable prognosis in lung cancer [158], making it an attractive target for therapeutic intervention [9,75]. Given the interplay between NRF2 and NOTCH signaling, alterations in these pathways have substantial therapeutic implications, particularly in terms of their prognostic value, utility as predictive biomarkers of treatment response, and overall impact on patient outcomes [11].

In lung cancer, genetic and epigenetic alterations affecting the NRF2 and NOTCH signaling pathways have emerged as key determinants of tumor behaviour and clinical outcome. Activating mutations in *NFE2L2* or inactivating mutations of its negative regulator *KEAP1* promote oxidative stress resistance, metabolic reprogramming, and immune evasion [56]. Similarly, mutations in *NOTCH* receptors, particularly missense variants affecting ligand-binding or ankyrin domains, can result in ligand-independent activation, contributing to altered cell fate decisions and suppression of tumor suppressor functions [2]. These molecular events are associated with poor prognosis, reduced OS and PFS, and resistance to conventional therapies, including chemotherapy, targeted therapies such as those with tyrosine kinase inhibitors (TKIs), and immunotherapy [201].

Recent genomic and functional studies have increasingly underscored the prognostic impact of alterations in the NRF2–NOTCH signaling on distinct lung cancer subtypes. Constitutive activation of the NRF2 pathway, mainly resulting from *KEAP1* loss-of-function or *NFE2L2* gain-of-function mutations, occurs in approximately 20–30% of NSCLCs, especially LUAD and LUSC, as previously reported [158]. In NSCLC, aberrant activation of NRF2 not only enhances oxidative stress resistance and therapeutic tolerance, but also modulates NOTCH1 signaling, influencing apoptotic responses following radiation exposure. This functional interaction contributes to therapy resistance and may define more aggressive disease phenotypes [202].

In large-cell neuroendocrine carcinomas (LCNECs), mutations in genes regulating NRF2 activity and members of the NOTCH receptor family are frequently observed, particularly in NSCLC-like LCNECs, where NOTCH pathway mutations help to discriminate them from classical adenocarcinomas [203]. Conversely, SCLC-like LCNECs show a higher incidence of *KEAP1* and *NFE2L2* mutations, uncommon in canonical SCLC but frequent in squamous cell carcinoma, suggesting divergent developmental trajectories influenced by these pathways [204]. Recently, high NRF2 expression in NSCLC patients were found to be correlated with poorer OS and PFS following chemotherapy or EGFR TKIs treatment, suggesting NRF2 as a potential marker of tumor aggressiveness and a valuable tool for prognosis and optimizing treatment strategies [205].

Moreover, pan-cancer analyses have revealed recurrent co-mutations in *KEAP1*, *NFE2L2*, and *NOTCH1*, supporting a cooperative role in driving tumor progression. These co-alterations may amplify oncogenic signaling and reinforce treatment resistance, thereby correlating with worse clinical outcomes [77,206].

Taken together, disruptions in the NRF2–NOTCH axis emerge as key prognostic indicators in lung cancer, with potential utility in molecular stratification and targeted therapeutic strategies. Moving into predictive insights, *KEAP1* and *NFE2L2*-*mutant* tumors exhibit reduced responsiveness to immune-checkpoint inhibitors (ICIs), likely due to an immunosuppressive microenvironment, characterized by low tumor mutational burden, reduced interferon signaling, and decreased CD8+ T cell infiltration. This may suggest an important predictive value of pharmacologic inhibition of NRF2 as a strategy to restore therapeutic sensitivity, which is currently under preclinical investigation [207].

Given its complex role in lung cancer, NOTCH signaling is emerging as a prognostic and predictive cancer biomarker, as its aberrant activation contributes to tumor progression, therapeutic resistance, and increased recurrence in both NSCLC and SCLC [2].

Clinical findings have shown that elevated NOTCH3 expression was a predictor of different aggressive tumor behaviours, such as advanced clinical stage and lymph node metastasis in a well-defined cohort of NSCLC patients [208] and, later, it has been found to be correlated with reduced sensitivity to platinum-based chemotherapy in NSCLC. These findings suggested a role for NOTCH3 in driving the advancement and chemoresistance of NSCLC and supporting the potential role of NOTCH3 as a predictive biomarker for treatment response in this cohort of patients [126].

Importantly, emerging data point to a context-dependent predictive role of NOTCH mutations in immunotherapy. Among NOTCH alterations, *NOTCH1* mutations have been linked to an increase in MHC class I expression and T-cell infiltration, two features that may sensitize NSCLC patients to ICIs, corroborating the idea that specific *NOTCH* mutations could serve as independent predictive biomarkers in the prognosis of NSCLC patients treated with ICIs [209].

By making tumor cells more susceptible to oxidative stress, NRF2 inhibitors, like Brusatol [210,211,212] and ML385 [213,214], have been studied for their capacity to prevent NRF2 activation and improve the efficacy of chemotherapy and radiation therapy [215,216]. Drugs targeting the NRF2 pathway also frequently block downstream antioxidant genes that support cancer cell survival or prevent the pathway’s interaction with *KEAP1*, a negative regulator of NRF2 [215].

Several approaches for the NOTCH pathway are being investigated. Gamma-secretase inhibitors (GSIs), such as DAPT [83,217] and RO4929097 [218], seem to be promising in preclinical studies for their ability to block the cleavage of NOTCH receptors, preventing downstream signaling activation [219]. These inhibitors, by disrupting the aberrant activation of NOTCH signaling, can arrest tumor growth in lung cancer cells. However, because of its intricate involvement in maintaining normal tissue homeostasis, NOTCH signaling has proven difficult to target, and the therapeutic window is still limited because of possible adverse consequences such as immune suppression [11].

Additional tactics include the use of small compounds to alter the way NOTCH interacts with its downstream effectors and monoclonal antibodies, as well as tarextumab [135], that target NOTCH receptors or ligands, with intriguing results [220]. NOTCH signaling inhibitors are often explored in combination with other therapies to improve treatment efficacy, particularly in NSCLC and SCLC [8]. NOTCH inhibitors, especially GSIs, are sometimes used in combination with standard chemotherapy or targeted therapies to overcome drug resistance. For example, blocking NOTCH signaling can make cancer cells more sensitive to traditional chemotherapy agents (like cisplatin) and targeted agents (like EGFRi) [221].

Since NOTCH signaling is involved in immune cell regulation and the maintenance of cancer stem cells, combining NOTCH inhibitors with immune checkpoint inhibitors (ICIs, such as anti-PD-1 or anti-PD-L1) has been explored. This strategy may enhance the immune response against lung tumors [222]. While these drugs are still being tested in clinical trials, they represent exciting prospects for enhancing lung cancer treatment.

Emerging data also highlight potential crosstalk between NRF2 and NOTCH signaling axes, which may synergistically drive tumor aggressiveness and resistance phenotypes, resulting in significant clinical implications as detailed in this section [223]. Given their multifaceted roles, integrative profiling of NRF2 and NOTCH alterations, at genomic, transcriptomic, and protein levels, could improve patient stratification and guide the development of combination strategies (e.g., NRF2 or NOTCH inhibitors with ICIs or standard therapies) aimed at overcoming resistance and improving durable clinical responses in lung cancer.

However, challenges such as toxicity, tumor heterogeneity, and resistance need to be addressed before these inhibitors can become mainstream treatments for lung cancer [224]. Table 4 summarizes the targeting of NRF2 and NOTCH pathways, their associated mechanisms of action, and relevant drugs.

## 10. Multi-Omics and Emerging Technologies: Current Advances and Future Directions

Emerging technologies, particularly single-cell multi-omics, are poised to significantly enhance the understanding of the NRF2–NOTCH signaling pathways, which are crucial in various biological processes and diseases. These technologies allow us to perform a detailed analysis of cellular heterogeneity, molecular interactions and functional states across tumor microenvironments [230]. In this context, single-cell RNA sequencing (scRNA-seq) and chromatin accessibility assays (scATAC-seq) hold great value and can be effectively applied to dissect NRF2- and NOTCH-driven transcriptional and epigenetic programs at a cell-specific level. These approaches are able to cover context-dependent interactions, such as NRF2 binding to AREs in regulatory regions of NOTCH1 or repression of NRF2 target genes by NOTCH effectors [231].

Although Chromatin immunoprecipitation sequencing (ChIP-seq) remains the gold standard for profiling chromatin-protein interactions, its high input material requirements pose significant challenges for applications at the single-cell level [232]. By profiling histone marks and transcription factor occupancy associated with NRF2, KEAP1, and NOTCH proteins, CUT&Tag (Cleavage Under Targets and Tagmentation) facilitates the identification of dynamic chromatin accessibility changes and regulatory element activity that govern their gene expression and functional status [230]. These approaches could be further strengthened by integrative proteogenomic techniques, such as Cellular Indexing of Transcriptomes and Epitopes by Sequencing (CITE-seq) and RNA Expression and Protein Sequencing (REAP-seq), single-cell multi-omics platforms that simultaneously quantify gene expression and surface protein levels within individual cells, providing a powerful means to dissect oxidative stress responses, immune modulation, and the regulatory dynamics underlying NRF2–NOTCH pathway crosstalk [233].

To contextualize NRF2–NOTCH pathway activity within the native tissue environment, spatial transcriptomics platforms such as 10× Genomics Visium and Slide-seq retain spatial information, enabling precise mapping of pathway activation and its interactions with stromal and immune components across distinct tumor niches [234]. Finally, functional studies employing perturbation sequencing, which combines Clustered Regularly Interspaced Short Palindromic Repeats (CRISPR)-mediated gene perturbations with single-cell transcriptomic readouts, have revealed critical vulnerabilities and adaptive dependencies that tumor cells acquire upon loss of *KEAP1*, *NFE2L2*, or *NOTCH1*, thereby uncovering potential targets for therapeutic intervention [235,236].

Despite their great potential, these technologies face key challenges, particularly in data integration and clinical translation, highlighting the need for standardized methods, longitudinal sampling, and validation in relevant models to effectively exploit single-cell multi-omics and identify therapeutic vulnerabilities in the NRF2–NOTCH axis in lung cancer.

## 11. Conclusions

The dynamic crosstalk between NRF2 and NOTCH signaling pathways plays a pivotal role in lung cancer biology, since their reciprocal regulation influences tumor initiation, progression, and therapy resistance [10].

Key takeaways from this review include: (1) NRF2 can transcriptionally activate NOTCH1, while NOTCH signaling regulates NRF2 activity via RBPJ, illustrating a bidirectional and context-specific interaction [11]; (2) both pathways can function as oncogenes or tumor suppressors, depending on tumor subtype and microenvironmental cues [7]; (3) genetic alterations, such as *KEAP1*, *NFE2L2* and *NOTCH1* mutations, significantly impact on their signaling networks [237]; (4) epigenetic mechanisms, including DNA methylation, histone modifications, and ncRNAs, add another layer of regulation, contributing to pathway deregulation in NSCLC [238]; (5) metabolic reprogramming, a hallmark of aggressive tumors, further modulates NRF2–NOTCH interactions, driving phenotype plasticity and drug resistance [169,186]; (6) therapeutic implications: advances in high-throughput and single-cell multi-omics technologies now allow for a more comprehensive analysis of the crosstalk between these pathways, paving the way for the development of NRF2–NOTCH inhibitors and the implementation of personalized therapeutic strategies [239].

Overall, understanding the intricate NRF2–NOTCH axis provides a promising framework for designing innovative strategies to improve therapeutic responses and clinical outcomes in lung cancer patients.

## Figures and Tables

**Table 1:** An overview of NRF2-KEAP1 pathway alterations in lung cancer, covering genetic mutations, epigenetic modifications, associated molecular mechanisms, and clinical implications.

Mechanism	Effect on NRF2-KEAP1 Pathway	Impact on Lung Cancer	Associated Molecular Mechanisms	Clinical Implications	**Ref.**
***KEAP1* mutation**	Lost of *NRF2-KEAP1* interaction due to LOF, causing the activation of NRF2	A cytoprotective mechanism triggered by NRF2 constitutive activation	*KEAP1* mutations are mainly located in the IVR and KELCH1-6 domains (e.g., p.D236H, p.R320Q)	Resistance to radiation, chemotherapy, or immunotherapy. More commonly observed in LUAD patients with poor prognosis	[56,57,58,59]
***NFE2L2* mutation**	Presence of GOF mutations in *NFE2L2*, leading to the stabilization of NRF2 and its nuclear accumulation	An increased NRF2 activity that contributes to stress resistance and promotes cancer cell survival	Alterations in the DLG and ETGE motifs, such as p.D29N and p.G81V, leading to KEAP1-dependent degradation of NRF2	An increased growth speed and invasiveness, chemoresistance (e.g., crizotinib), and worse clinical outcomes in NSCLC patients	[62]
***KEAP1* aberrant promoter methylation**	Silencing of *KEAP1* gene expression, leading to NRF2 activation	An increased NRF2 expression	DNA methylation at CpG sites within *KEAP1* promoter region reduces its expression levels	A potential utility as prognostic biomarker in targeting epigenetic therapy; for instance, DNMT inhibitors (e.g., decitabine) may rescue KEAP1 expression by impairing NRF2 activation	[81,83,84,85,87]
**NRF2-KEAP1 histone modifications**	Repression of NRF2-KEAP1 expression	An impaired NRF2 signaling contributes to cancer cell survival and proliferation	NRF2 expression can be suppressed by *EZH2*-mediated mechanism (H3K27me3 marks at its promoter)	Histone deacetylation may restore NRF2 repression, enhancing effects on therapeutic efficacy	[87]
**KEAP1 downregulation via miRNAs**	A reduced KEAP1 activity implies a dysregulated NRF2 signaling	Rapid growth, survival, and ability to switch their metabolic pathways	miR-200a, miR-141, and miR-140 directly inhibit KEAP1 translation	Small molecule-mediated miRNA modulation may be a viable way for therapeutic strategy	[93]
**NRF2-regulated miRNA expression**	NRF2 constitutive activation impairs miRNA expression levels, thereby reinforcing its pathway	An increased cellular susceptibility to oxidative stress with a considerable impact on metabolism phenotype	miR-1 and miR-206 modulate the pentose phosphate pathway, leading to abnormal cell proliferation	Targeting miRNA molecules as a promising and effective approach for combined epigenetic and metabolic therapy	[94]
**Long non-coding RNAs**	SCAL1 and NLUCAT1 mediate oxidative stress through NRF2 activation	Short isoform SCAL1 induced by cigarette smoke extracts in NSCLC cell lines; NLUCAT1 is upregulated in hypoxic conditions in a subset of LUAD cell lines	The upregulation of SCAL1 is linked to an increase in sensitivity of oxidative stress response; LUCAT1 transcripts were found to be associated with *KEAP1* and *NFE2L2* mutations in LUADs and LUSCs	Targeting SCAL1 and NLUCAT1 lncRNAs represent a promising therapeutic strategy to overcome chemoresistance and improve treatment outcomes in lung cancer patients	[101,102]

Abbreviations: KEAP1, Kelch-like ECH-associated protein 1; NRF2, Nuclear factor erythroid 2-related factor 2; IVR, Intervening Region; KELCH, Kelch domain; LUAD, Lung Adenocarcinoma; LOF, Loss of Function; GOF, Gain of Function; DLG, Asp-Leu-Gly motif; ETGE, Glu-Thr-Gly-Glu motif; NSCLC, Non-Small-Cell Lung Cancer; DNMT, DNA Methyltransferase; *EZH2*, Enhancer of Zeste Homolog 2; H3K27me3, Trimethylation of lysine 27 on histone H3; miR, MicroRNA; SCAL1, Smoke and Cancer-Associated Long non-coding RNA 1; lncRNAs, Long non-coding RNAs; NLUCAT1, Non-coding Lung Cancer Associated Transcript 1.

**Table 2:** Overview of alterations in NOTCH signaling in lung cancer, focusing on mutations, epigenetic modifications, and molecular mechanisms, along with their potential clinical implications.

Mechanism	Effect on NOTCH Signaling	Impact on Lung Cancer	Associated Molecular Mechanisms	Clinical Implications	**Ref.**
***NOTCH1* mutation**	GOF mutations could promote the activation of canonical NOTCH signaling pathway	An increased stemness properties, tumor growth and drug resistance mechanism	Missense, synonymous, and silent mutations in *NOTCH* functional domains (e.g., EGF-like, LNR, Ankyrin repeats, PEST)	Worse prognosis, especially in *TP53-mutated* tumors with a poor response to anticancer therapy	[138]
***NOTCH1* epigenetic regulation (via *NUMB*)**	A reduced NUMB expression contributes to NOTCH1 activation	Cancer stem cell characteristics as well as enhanced clonal growth and drug resistance	*NUMB* promoter hypermethylation can lead to NOTCH1 transcriptional silencing	The potential of using epigenetic modifications as diagnostic biomarkers and therapeutic targets	[139]
***NOTCH1* promoter methylation**	Stabilization of DNMT1 occurs as a consequence of the silencing of NOTCH1 expression	NOTCH signaling impedes apoptosis and accelerates proliferation with an inhibition of tumor growth	DNMT1 stabilizes *NOTCH1* promoter methylation, leading to the disruption of its tumor-suppressive functions	Targeting epigenetic regulators with DNMT inhibitors in order to reverse gene silencing	[145,146]
**NOTCH1 regulation by miRNAs**	miRNAs mediated repression of NOTCH1 as a critical post-transcriptional regulatory mechanism	Microenvironment influence on cell fate decision in terms of migration, and differentiation	miR-34a, miR-200, and other miRNAs modulate NOTCH signaling by fine-tuning its activity at multiple levels of this pathway	Inhibitors of miRNAs can directly regulate the biological behavior of lung cancer cells and control their progression	[147,148]
**NOTCH1/NOTCH3 regulation by lncRNAs**	lncRNAs regulate the NOTCH pathway through miRNA sequestration	An acceleration in tumor cell proliferation, migration, and invasion	lncRNA AGAP2-AS1 and SNHG11 act as competing endogenous RNAs that sponge miRNAs and prevent them from suppressing NOTCH pathway components	lncRNA-targeted therapies represent a powerful strategy for inhibiting NOTCH-driven tumor progression	[149,150,151]
**NOTCH1 activation via lncRNA PVT1**	The upregulation of PVT1 leads to the activation of NOTCH1	Promotion of oncogenic processes such as cell proliferation, EMT, and metastasis	The intersection between the activation of PVT1-EZH2 axis and the suppression of miR-497 contributes to NOTCH1 upregulation	Silencing of *PVT1* may serve as a therapeutic strategy to block growth and metastasis formation of lung cancer	[152]

Abbreviations: NOTCH1, Neurogenic locus NOTCH homolog protein 1; GOF, Gain of Function; EGF-like, Epidermal Growth Factor-like repeats; LNR, LIN-12 repeat; PEST, Proline (P), Glutamic acid (E), Serine (S), and Threonine (T)-rich sequence; TP53, Tumor Protein p53; NUMB, NUMB Endocytic Adaptor Protein; DNMT1, DNA methyltransferase 1; miRNA, microRNA, NOTCH3, Neurogenic locus NOTCH homolog protein 3; lncRNAs, Long Non-Coding RNAs; AGAP2-AS1, AGAP2 Antisense RNA 1; SNHG11, Small Nucleolar RNA Host Gene 11; PVT1, Plasmacytoma Variant Translocation 1; EMT, Epithelial-to-Mesenchymal Transition; EZH2, Enhancer of Zeste Homolog 2.

**Table 3:** Main metabolic mechanisms modulated via NRF2–NOTCH axis in lung cancer.

Metabolic Mechanism	Key Findings	**Ref.**
**NRF2 and redox homeostasis**	NRF2 directly regulates ROS levels by activating enzymes like NQO1, GCLM, and HMOX1 to maintain intracellular redox homeostasis	[174,175]
**NRF2 and NADPH production**	NRF2 upregulates a set of enzymes (G6PD, IDH1, ME1, and PGD) involved in NADPH production, controlling redox balance, biosynthetic processes and host defense	[94,179,180,200]
**NRF2 and glycolysis**	NRF2 constitutive activation is linked to an increased of lactate production and glucose uptake, leading to tumor aggressiveness and metastasis in lung cancer	[183]
**NRF2 and glutamine metabolism**	NRF2 modulates glutamine metabolism by overexpressing key enzymes as well as GLS, GOT2, and MDH2, sustaining TCA cycle and reductive carboxylation	[187]
**NRF2 and glutathione**	NRF2 supports the synthesis of GSH by enhancing GCL and GSS, improving antioxidant defenses and leading to the resistance against chemo- and radiotherapy	[188,189,190]
**NOTCH and glycolysis**	NOTCH signaling upregulates MYC and promotes a metabolic shift towards a glycolytic phenotype in a tissue-specific manner, with a rapid acceleration in tumor growth and metastasis	[173]
**NOTCH and serine biosynthesis**	NOTCH increases the expression of enzymes such as ALDOA, GAPDH, PSAT1, and SHMT2, facilitating nucleotide synthesis and bolster antioxidant defense	[186]
**NOTCH and glutaminolysis**	NOTCH enhances GLS, GOT2, and MDH2 levels, targeting glutaminolysis in order to sustain the cancer metabolism and the functional role of TCA cycle intermediates	[187]
**NRF2 and lipid metabolism**	NRF2 inhibits de novo lipogenesis and participates in FAO, supporting energy production under stress conditions	[179,191]
**NRF2 and ferroptosis resistance**	NRF2 regulates ferroptosis by controlling *SLC7A11* and *GPX4* genes, hampering lipid peroxidation and resisting to apoptosis under oxidative stress	[193,194]
**NRF2 and iron homeostasis**	NRF2 promotes iron homeostasis by activating FTH1 and HMOX1, which is involved in protecting cells from ferroptosis	[197]
**NRF2, BACH1 and metastasis**	Stabilization of BACH1, driven by NRF2 activation, promotes metastasis through the upregulation of glycolytic genes as well as HK2 and GAPDH	[183]
**NRF2 and chemoresistance**	NRF2 orchestrates ROS balance and contributes to chemoresistance by activating PI3K/Akt pathway	[178]
**NRF2 and tumor metabolic phenotype**	NRF2 shapes metabolic phenotype in lung adenocarcinoma with a high glycolytic activity, and it is associated with poor prognosis and metastatic potential	[184]

Abbreviations: NRF2, Nuclear factor erythroid 2-related factor 2; ROS, Reactive Oxygen Species; NQO1, NAD(P)H quinone dehydrogenase 1; GCLM, Glutamate-cysteine ligase modifier subunit; HMOX1, Heme oxygenase 1; G6PD, Glucose-6-phosphate dehydrogenase; IDH1, Isocitrate dehydrogenase 1; ME1, Malic enzyme 1; PGD, 6-phosphogluconate dehydrogenase; NADPH, Nicotinamide adenine dinucleotide phosphate; GLS, Glutaminase; GOT2, Glutamic-oxaloacetic transaminase 2; MDH2, Malate dehydrogenase 2; TCA, Tricarboxylic acid; GSH, Glutathione; GCL, Glutamate-cysteine ligase; GSS, Glutathione synthetase; MYC, Myelocytomatosis oncogene; ALDOA, Aldolase A; GAPDH, Glyceraldehyde-3-phosphate dehydrogenase; PSAT1, Phosphoserine aminotransferase 1; SHMT2, Serine hydroxymethyltransferase 2; GLS, Glutaminase; GOT2, Glutamic-oxaloacetic transaminase 2; MDH2, Malate dehydrogenase 2; FAO, Fatty acid oxidation; SLC7A11, Solute carrier family 7 member 11; GPX4, Glutathione peroxidase 4; FTH1, Ferritin heavy chain 1; HMOX1, Heme oxygenase 1; BACH1, BTB and CNC homology 1; HK2, Hexokinase 2; GAPDH, Glyceraldehyde-3-phosphate dehydrogenase; PI3K/Akt, Phosphoinositide 3-kinase/Protein kinase B.

**Table 4:** Drugs targeting NRF2 and NOTCH pathways and related mechanisms of action.

	Target Pathway	Mechanism of Action	Development Stage	Comments	**Clinical Trial**	**Ref**
**Brusatol**	NRF2	Inhibits the NRF2 transcription factor, sensitizing cells to chemotherapy and radiotherapy	Preclinical research	Investigational small molecule studied to inhibit NRF2, enhancing the sensitivity to therapies in solid cancer	N/A	[210,211,212]
**ML385**	NRF2	Inhibits NRF2 activity, reducing antioxidant response and increasing susceptibility to oxidative stress	Preclinical research	Investigational NRF2 inhibitor that sensitizes cells to oxidative stress, improving the effectiveness of chemotherapy and radiotherapy in various lung cancer cells	N/A	[213,214]
**DAPT**	NOTCH	Gamma-secretase inhibitor that blocks the cleavage of NOTCH receptors, preventing downstream signaling activation	Preclinical research	Suppresses NOTCH signaling, arresting tumor growth in solid cancer including SCLC cells	N/A	[83,217]
**MK-0752**	NOTCH	Gamma-secretase inhibitor that blocks NOTCH receptor activation	Clinical studies	Investigational drug in clinical trials for cancer	NCT01098344, NCT01295632	[225,226]
**RO4929097**	NOTCH	Selective gamma-secretase inhibitor that blocks NOTCH receptor activation	Clinical studies	Investigational inhibitor in clinical trials for solid tumors	NCT01149356, NCT01120275	[218,227]
**NAM**	NOTCH	Modulates NOTCH signaling and has the potential as a NOTCH pathway inhibitor	Preclinical research	Preclinical studies on the effect of nicotinamide in influencing NOTCH signaling	N/A	[228]
**Nelfinavir**	NOTCH	HIV protease inhibitor that has off-target effects inhibiting NOTCH signaling in tumor cells	Preclinical research	HIV protease inhibitor with emerging evidence to inhibit NOTCH signaling in certain cancers	N/A	[229]
**Tarextumab**	NOTCH	Monoclonal antibody targeting NOTCH2, used in treating cancers with activated NOTCH	Clinical studies	Investigational drug targeting NOTCH2 in various cancers, including SCLC	NCT01859741	[135]

Abbreviations: NRF2, Nuclear Factor Erythroid 2-Related Factor 2; NOTCH, Neurogenic Locus NOTCH Homolog; NSCLC, Non-Small-Cell Lung Cancer; NAM, Nicotinamide; HIV, Human Immunodeficiency Virus; NOTCH2, Neurogenic Locus NOTCH Homolog 2; SCLC, Small-Cell Lung Cancer. N/A, not available.
